# Alkylation of Histidine Residues of *Bothrops jararacussu* Venom Proteins and Isolated Phospholipases A_2_: A Biotechnological Tool to Improve the Production of Antibodies

**DOI:** 10.1155/2014/981923

**Published:** 2014-05-11

**Authors:** C. L. S. Guimarães, S. H. Andrião-Escarso, L. S. Moreira-Dill, B. M. A. Carvalho, D. P. Marchi-Salvador, N. A. Santos-Filho, C. A. H. Fernandes, M. R. M. Fontes, J. R. Giglio, B. Barraviera, J. P. Zuliani, C. F. C. Fernandes, L. A. Calderón, R. G. Stábeli, F. Albericio, S. L. da Silva, A. M. Soares

**Affiliations:** ^1^Center of Applied Biomolecules Studies in Health (CEBio), Oswaldo Cruz Foundation (Fiocruz Rondônia), Porto Velho, RO, Brazil; ^2^Department of Medicine, Federal University of Rondônia (UNIR), Porto Velho, RO, Brazil; ^3^Brazilian Institute of Environment and Renewable Natural Resources (IBAMA), Porto Velho, RO, Brazil; ^4^Department of Biochemistry and Immunology, Faculty of Medicine of Ribeirão Preto, University of São Paulo (USP), Ribeirão Preto, SP, Brazil; ^5^Department of Chemistry, Biotechnology and Bioprocess Engineering, Federal University of São João Del Rei (UFSJ), Ouro Branco, MG, Brazil; ^6^Department of Molecular Biology, Center for Science and Nature, Federal University of Paraíba (UFPB), João Pessoa, Brazil; ^7^Department of Clinical Analysis, Faculty of Pharmaceutical Sciences of Ribeirão Preto, University of São Paulo (USP), Ribeirão Preto, SP, Brazil; ^8^Department of Physics and Biophysics, State University Paulista (UNESP), Botucatu, SP, Brazil; ^9^Center for the Study of Venoms and Venomous Animals (CEVAP), State University Paulista (UNESP), Botucatu, SP, Brazil; ^10^Institute for Research in Biomedicine (IRB Barcelona) and CIBER-BBN, Barcelona Science Park, Barcelona, Spain; ^11^Department of Organic Chemistry, University of Barcelona, Barcelona, Spain; ^12^School of Chemistry and Physics, University of KwaZulu Natal, Durban 4001, South Africa

## Abstract

Crude venom of *Bothrops jararacussu* and isolated phospholipases A_2_ (PLA_2_) of this toxin (BthTX-I and BthTX-II) were chemically modified (alkylation) by *p*-bromophenacyl bromide (BPB) in order to study antibody production capacity in function of the structure-function relationship of these substances (crude venom and PLA_2_ native and alkylated). BthTX-II showed enzymatic activity, while BthTX-I did not. Alkylation reduced BthTX-II activity by 50% while this process abolished the catalytic and myotoxic activities of BthTX-I, while reducing its edema-inducing activity by about 50%. Antibody production against the native and alkylated forms of BthTX-I and -II and the cross-reactivity of antibodies to native and alkylated toxins did not show any apparent differences and these observations were reinforced by surface plasmon resonance (SPR) data. Histopathological analysis of mouse gastrocnemius muscle sections after injection of PBS, BthTX-I, BthTX-II, or both myotoxins previously incubated with neutralizing antibody showed inhibition of the toxin-induced myotoxicity. These results reveal that the chemical modification of the phospholipases A_2_ (PLA_2_) diminished their toxicity but did not alter their antigenicity. This observation indicates that the modified PLA_2_ may provide a biotechnological tool to attenuate the toxicity of the crude venom, by improving the production of antibodies and decreasing the local toxic effects of this poisonous substance in animals used to produce antivenom.

## 1. Introduction

Snakebites are a public health problem in tropical countries as they can lead to death or serious sequelae. For example, in Brazil, the Viperidae family has four genera of venomous snake, the* Bothrops* genus being responsible for 87.5% of incidents reported, followed by* Crotalus *(9.2%),* Lachesis* (2.7%), and snakes that belong to the Elapidae family (0.6%) [1].

*Bothrops* venom is rich in phospholipases A_2_ (PLA_2_), a class of enzymes with catalytic activity on cell membranes of specific tissues. This observation would suggest that PLA_2_ plays a crucial role in venom toxicity [2, 3]. Furthermore, snake venom PLA_2_ induces several other effects such as pre- or postsynaptic neurotoxicity [3–5], cardiotoxicity [6–9], myotoxicity [10, 11], platelet aggregation inhibition or induction [12, 13], edema [14–17] and hypotension [18–20]. Thus, medical and scientific interest in PLA_2_s lies in the action of these enzymes in various pathophysiological processes. In this regard, several research lines are devoted to searching for drugs or tools to inhibit or neutralize the action of PLA_2_.

The development of natural or synthetic inhibitors with the capacity to neutralize the toxic effects of PLA_2_s has advanced our medical knowledge of the mechanisms of action and structure-function relationships of these enzymes. In this respect, our research group focuses on the development of biotechnological tools that inhibit the PLA_2_s and its toxic effects [21, 22].

Snake venom PLA_2_s belong to classes I or II of this enzyme family. In this regard, the PLA_2_ from venom of* Bothrops jararacussu* belongs to the latter [2–4, 22]. The enzymatic activity of the PLA_2_s is characterized by the integrity of the amino acid residue His48 in the active site.

The literature describes that PLA_2_ loses its catalytic activity when His48 undergoes alkylation by* p*-bromophenacyl bromide (BPB). In addition to this effect on the activity of PLA_2_s, the alkylation also leads to a reduction in the toxic and pharmacological activity of this enzyme [23].

The clinical treatment for* Bothrops* snake bites basically consists of the early administration of appropriate doses of anti-Bothropic serum [24]. The production of antivenom, usually conducted with horses, involves the subcutaneous injection of increasing amounts of the corresponding venoms [25, 26]. Although these injections do not cause relevant systemic alterations in the animals, they induce significant tissue damage at the site of injection (edema, hemorrhage, and necrosis). Reduction of local tissue reaction at the site of inoculation, without impairing the immune response of the animals against venom components, is a major goal of laboratories producing antivenoms.

Here, we report on the development of a biotechnology tool based on native BjussuCV (*B. jararacussu* crude venom), BthTX-I (K49-PLA_2_-like) and BthTX-II (D49-PLA_2_), and also all proteins alkylated by BPB. The enzymatic and biological activities of BjussuCV, BthTX-I, and BthTX-II in their native and alkylated forms were evaluated and compared. Rabbit polyclonal antibodies against native or chemically modified BjussuCV, BthTX-I, and -II were produced, and assays were performed to assess their capacity to neutralize the phospholipasic, myotoxic, edema-inducing, and lethal activities of their corresponding antigens. Our results identify the modified toxins as potential tool to produce antivenoms and simultaneously diminish the local effects of injections on animals used to produce antivenom, without impairing their immune response.

## 2. Material and Methods

### 2.1. Venoms and Chemicals

The* B. jararacussu* snake venom pool, collected in São Paulo state (*n* = 6), was provided by L. H. A. Pedrosa (FMRP-USP). The proteins BthTX-I (K49-PLA_2_-like) and BthTX-II (D49-PLA_2_) were isolated as previously described [27, 28]. The venom (BjussuCV) was dried over NaOH pellets in a vacuum desiccator at room temperature immediately after collection and stored at 4°C.

Chemicals such as acrylamide, bisacrylamide, TEMED, Coomassie Brilliant Blue G-250,* p*-bromophenacyl bromide, sinapinic acid (Sigma), bromophenol Blue (Merck), mercaptoethanol (Fluka AG), CM-Sepharose (Pharmacia), pH 5.0–8.5 buffalyte (Pierce), and Freund adjuvant were of analytical grade.

### 2.2. Animals

Male Swiss mice (18–22 g) and rabbits (2.0–2.5 kg) were obtained from the Central Bioterium of S. Paulo University, Ribeirão Preto, SP. Animal care was in accordance with the guidelines of the Brazilian College for Animal Experimentation (COBEA), and the present proposal was approved by the Animal Ethics Committee of S. Paulo University (CEUA-USP) and The Brazilian Institute of Environment (IBAMA).

### 2.3. Alkylation of BjussuCV, BthTX-I, and BthTX-II

The BjussuCV, BthTX-I, and BthTX-II samples (12 mg) were dissolved in 1 mL of 100 mM ammonium bicarbonate buffer, pH 7.8, containing 0.7 mM EDTA. Then, 500 *μ*L of BPB (1.5 mg/mL ethanol) was added, and the samples were incubated at 25°C for 24 h. Excess reagent was removed by ultrafiltration through an Amicon YM3000 membrane, and the remaining protein solution was lyophilized [27, 28]. Native proteins are named BjussuCV, BthTX-I, BthTX-II, the proteins alkylated by BPB alk-BjussuCV, alk-BthTX-I, and alk-BthTX-II.

### 2.4. Immunization

1.6 mg of native or alkylated BjussuCV samples, as well as 0.5 mg of native or alkylated BthTX-I and BthTX-II, was dissolved in 1 mL of phosphate-buffered saline (PBS), to which 1 mL of Freund's complete adjuvant was added. Rabbits were then injected with 0.4 mL of these mixtures at four sites on the back and thighs (s.c.). After 12 days, a second immunization at the same sites was performed using Freund's incomplete adjuvant. Finally, after 45 days, a first bleed was carried out by intracardiac puncture. Normal and immune sera were precipitated with a saturated solution of ammonium sulphate, pH 6.5, left to stand for 30 min at 4°C, and centrifuged at 3800 ×g. The precipitate obtained was washed with 40% ammonium sulfate, centrifuged, dialyzed against 20 mM sodium phosphate buffer, pH 7.5, and concentrated through an Amicon YM10,000 membrane. Antibody purification was achieved by ion exchange chromatography on a 2 × 20 cm DEAE-Sephacel column, using 0.02 M sodium phosphate buffer, pH 7.5. Fractions of 4 mL/tube were collected by monitoring the absorbance at *λ* = 280 nm. Those fractions containing immunoglobulin G were pooled, concentrated through an Amicon YM10,000 membrane, lyophilized, and stored at 4°C until use [28].

### 2.5. Immunochemical Characterization

#### 2.5.1. Immunodiffusion

Rabbit polyclonal antibodies to native and alkylated proteins were tested against crude venom or isolated PLA_2_s (BthTX-I and BthTX-II) by gel immunodiffusion [29]. Twenty microliters of crude venom or PLA_2_ solutions (1.0 mg/mL PBS) and 20 *μ*L of antiserum were placed in wells with 1% agarose dissolved in PBS. The plates were put in a moist chamber at room temperature for 48 h for development of the precipitation lines and then washed for 24 h with 0.3 M NaCl. Experiments were performed in duplicate.

#### 2.5.2. Enzyme Immunoassays

Microplate wells were coated with BjussuCV, BthTX-I, and BthTX-II at 0.2 *μ*g/well by overnight incubation in 0.1 M Tris, 0.15 M NaCl, pH 9.0, buffer [30]. After five washes with solution A (50 mM Tris, 150 mM NaCl, 20 mM MnZnCl_2_, 1 mM MgCl_2_, and pH 7.4), the plates were air-dried and stored at 4°C. Purified rabbit antibodies against native (BjussuCV, BthTX-I, and BthTX-II) and modified proteins (alk-BjussuCV, alk-BthTX-I, and alk-BthTX-II) were diluted in solution A (containing 2% bovine serum albumin (BSA)), added to the microplate wells, and incubated at room temperature for 2 hours. After five washes, bound antibodies were detected with anti-rabbit immunoglobulin conjugated to alkaline phosphatase (Sigma), diluted 1 : 2000 with same solution A, and incubated for 90 minutes. After this time, the microplates were washed and the color was developed with* p*-nitrophenylphosphate. Absorbance was recorded with a microplate reader (BioTek) at 410 nm. Normal rabbit serum was used as a negative control in the place of the primary antibody. Assays were done in triplicate.

### 2.6. Inhibition of Biological Activities

Crude venom or native PLA_2_s and also crude venom or PLA_2_ alkylated by BPB were selected after dose-response studies of biological activities. The inhibition caused by antibodies was evaluated by incubation with each toxin: antibody ratios (1 : 10; 1 : 50, or 1 : 100) (w/w) for 30 min at 37°C. Biological activities were expressed as percentage, where 100% corresponded to activity of BjussuCV, BthTX-I, BthTX-II, alk-BjussuCV, alk-BthTX-I, and alk-BthTX-II incubated without antiserum.

#### 2.6.1. Myotoxic Activity

The creatine kinase (CK) assay was carried out using the CK-UV kinetic kit from Sigma. Assays were performed by injecting 50 *μ*L (i.m.) of solutions containing 1-2 *μ*g/*μ*L of venom or PLA_2_ (native and alkylated) dissolved in PBS, in the right gastrocnemius muscle of a group (*n* = 6) of male Swiss mice (18–22 g). The control group received only PBS. After 3 h, blood from the tail was collected in heparin-coated tubes and centrifuged for plasma separation. The amount of CK was then determined using 4 *μ*L of plasma, which was incubated for 3 min at 37°C with 1.0 mL of the reagent, following the manufacturer's instructions. Activity was expressed in U/L, where one unit resulted from the phosphorylation of 1 *μ*mol of creatine/min at 37°C [28, 29, 31–34].

#### 2.6.2. Edema-Inducing Activity

Groups of six Swiss male mice (18–22 g) were injected in the subplantar region of the right paw with 50 *μ*L of the native or modified toxins dissolved in saline solution (50 *μ*g/50 *μ*L). Paw edema was measured with a low pressure pachymeter (Mitutoyo, Japan) after 0.5, 1, and 3 h. Values registered at zero time point (measured before injections) were then subtracted from those obtained after injection, and the differences were reported as median% ± S.D. The minimal edematogenic dose (MED) was defined as the amount of venom or toxin (native or modified) that induced 30% edema in the paw at 0.5 h [27, 28, 34–37].

#### 2.6.3. Lethality (LD_50_)

Solutions differing in their concentration of native and modified proteins dissolved in saline solution were injected (i.p.) in a group (*n* = 6) of Swiss male mice (18–20 g). Each injection contained 100 *μ*L of solution. After injection, the mice were monitored for 24 h and values of lethal dose causing death of 50% of animals were calculated by Probitos method [38, 39].

#### 2.6.4. Enzymatic Activities

The phospholipase activity of the BjussuCV, BthTX-I, BthTX-II, alk-BjussuCV, alk-BthTX-I, and alk-BthTX-II (native and modified proteins) was assayed by indirect hemolytic activity, as previously described [40–42]. The clotting time test of citrated bovine plasma was used to determine coagulant activity. The minimal coagulant dose (MCD) is the amount of venom that induces coagulation in 0.2 mL of citrated bovine plasma in 60 sec at 37°C.

### 2.7. Protein Interactions

Protein-protein interactions were assayed by surface plasmon resonance (SPR) with a BIAcoreT200 system [43]. Proteins BthTX-I in Fc (Flow cell, Fc = 1) and BthTX-II in (Fc = 2) were covalently immobilized on the Serie S GE-BIAcore CM-5 sensorchip; they have carboxymethylated dextran matrix, according to the manufacturer's instructions, and the flow cells Fc = 3 and Fc = 4 were the blank (negative control). The flow cells Fc = 3 and Fc = 4 were the blanks (negative control). The CM-5 chip was activated with a 1 : 1 mixture of 0.4 M EDC (1-ethyl-3-(3-dimethylaminopropyl) carbodiimide) and 0.1 M NHS (1-hydroxy-2,5-pyrrolidinedione) for 5 minutes. Proteins were dissolved in 10 mM phosphate buffer, pH 5 (BthTX-I), and acetate buffer pH 5.9 (BthTX-II) and were then injected over the activated CM-5 chip at 25°C. The matrix was treated with 1 M ethanolamine/HCl pH 8.5 to block the remaining protein activated. Sample analyses were performed at a flow rate of 30 *μ*L/min for 1 min at 37°C. All results were analyzed using BIAevaluation software (version 1.1.1) [44]. Individual experiments were carried out three times.

### 2.8. Statistical Analysis

Results are presented as the mean ± S.D. obtained with the indicated number of animals. For statistical significance, the data were analyzed by Student's unpaired *t*-test, *P* < 0.05.

## 3. Results

**Figure 1:**
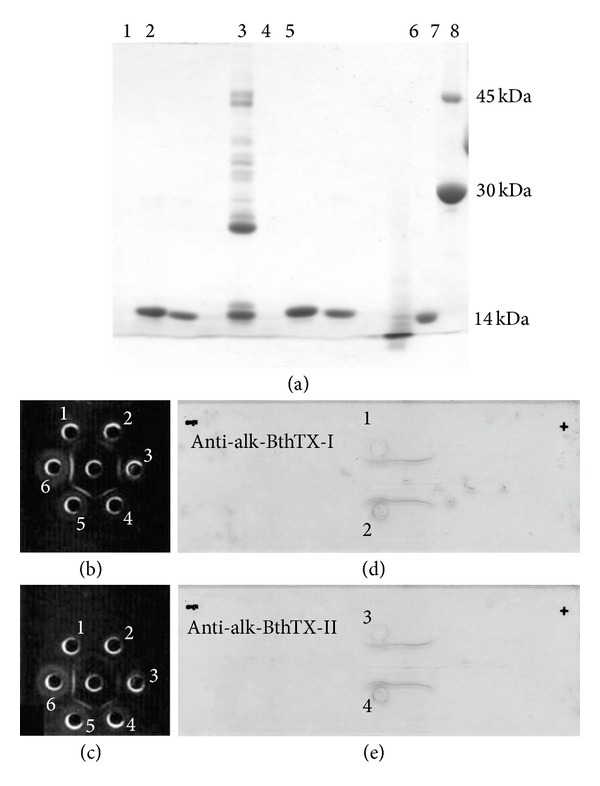
Electrophoretic and immunochemical analysis of the antigen-antibody reaction. (a) SDS-PAGE with reducing agents: lane 1, BthTX-I; lane 2, BthTX-II; lane 3, BjussuCV; lane 4, alk-BthTX-I; lane 5, alk-BthTX-II; lane 6, aprotinin (9 kDa); lane 7, *α*-lactalbumin (14 kDa); lane 8, carbonic anhydrase (30 kDa), and albumin egg (45 kDa). Antigen immunodiffusion: (b) native BthTX-I (central well) against its native anti-BthTX-I (1, 2, and 3 wells) and BPB-modified (4, 5, and 6 wells) antibodies; (c) native BthTX-II (central well) against its native anti-BthTX-II (1, 2, and 3 wells), and BPB-modified (4, 5, and 6 wells) antibodies. The precipitation lines were achieved using immunodiffusion gel in a wet chamber at 25°C for 48 h. Antigen immunoelectrophoresis: (d) anti-alk-BthTX-I (anti-BthTX-I-BPB) (central well) against its BthTX-I (1) and alk-BthTX-I (2) antigens; (e) anti-alk-BthTX-II (anti-BthTX-II-BPB) antibodies (central well) against its BthTX-II (3) and alk-BthTX-II (4) antigens. Results are representative of experiments carried out in triplicate.

*B. jararacussu* venom (BjussuCV) presented a chromatographic profile on CM-Sepharose column similar to that reported in previous studies [27]. BthTX-I and BthTX-II accounted for about 25% and 8% (w/w) of crude dried venom, respectively. The homogeneity of these proteins was verified by isoelectrofocusing and RP-HPLC. The electrophoretic profile in SDS-PAGE showed that alk-BthTX-I and alk-BthTX-II did not present differences when compared with the native BthTX-I and BthTX-II forms (Figure 1(a)).

Immunodiffusion and immunoelectrophoresis assays carried out with BthTX-I and BthTX-II (Figures 1(b)–1(e)) showed that a sufficient amount of antibody was produced after a 6-week period, after administration of the first dose of the antigen to the rabbits. This was verified by precipitation lines formed between native toxins (antigens) and their correspondent antibodies.

The enzyme immunoassays demonstrated that rabbit serum obtained after immunization with BjussuCV, BthTX-I, BthTX-II, alk-BjussuCV, alk-BthTX-I, and alk-BthTX-II contained antibodies that recognized the native and modified protein. The titration curves of antibodies against native and modified BthTX-I and BthTX-II revealed a moderate similarity. The lethality (LD_50_) and phospholipasic activity of native or modified protein were significantly reduced after alkylation (Table 1).

The enzymatic activity of PLA_2_ is dependent on the presence of a His residue in position 48. BthTX-I did not show enzymatic activity because this protein has a Lys in this position, thus removing the activity of enzyme. Neither BthTX-I nor alk-BthTX-I showed PLA_2_ activity. In addition, as expected, alk-BjussuCV and alk-BthTX-II suppressed the PLA_2_ activity of this enzyme.

We found that alk-BthTX-I and alk-BthTX-II reduce the lethal dose (LD_50_) relative to that of BthTX-I and BthTX-II. However, the alkylation of the crude venom did not cause a reduction in mortality relative to the native form. This observation could be attributable to the presence of other toxic proteins present in crude venom, such as metalloproteases and serinoproteases (Table 1).

**Figure 2:**
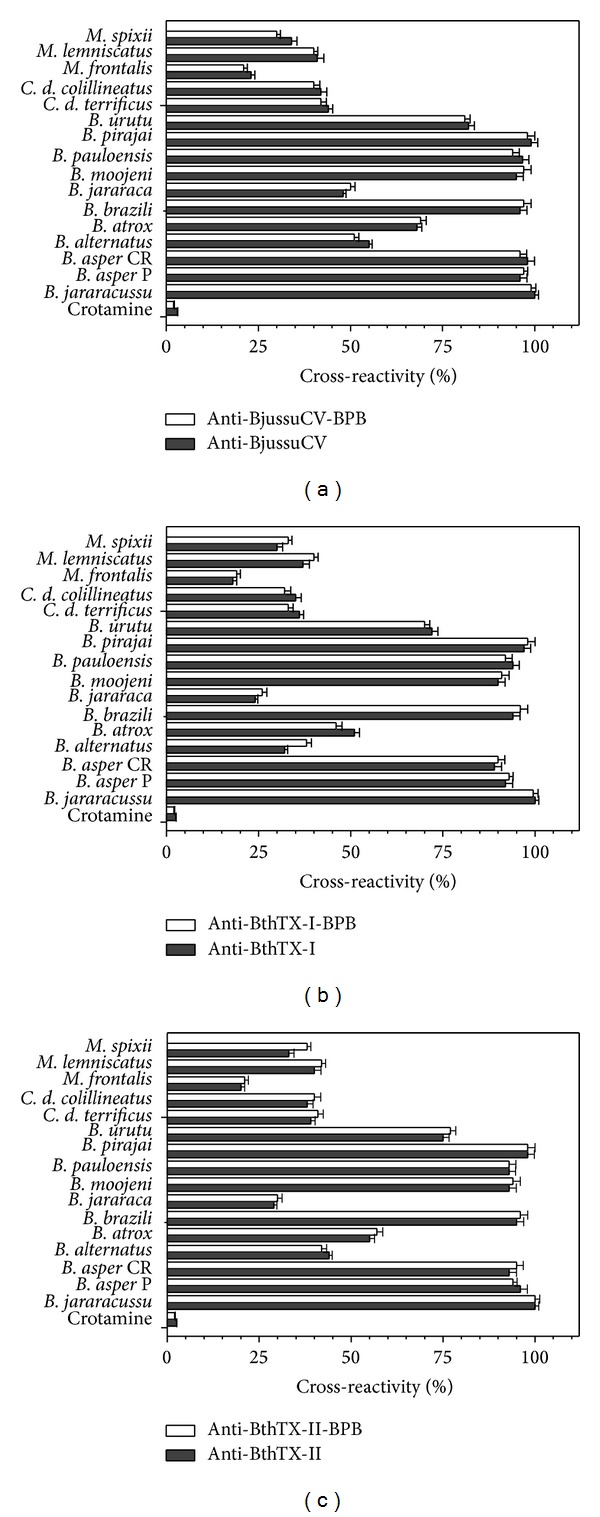
Cross-reactivity of antibodies produced from native or alkylated proteins from* B. jararacussu* and BthTX-I and BthTX-II (anti-BjussuCV, anti-alk-BjussuCV, anti-BthTX-I, anti-BthTX-I or anti-BthTX-II, and anti-alk-BthTX-II) against snake venoms of the* Bothrops*,* Crotalus,* and* Micrurus* genera, as shown by enzyme immunoassay. Microplate wells were coated with antigen, and antibody binding was measured, as described in Section 2.5.2. Cross-reactivity was expressed as a percentage of the absorbance signal resulting from the binding of antibodies to the homologous antigen ((a)* B. jararacussu* crude venom; (b) BthTX-I; (c) BthTX-II). Crotamine was included as an antigenically unrelated, negative control antigen. Results expressed as means ± S.D. (*n* = 3).

**Figure 3:**
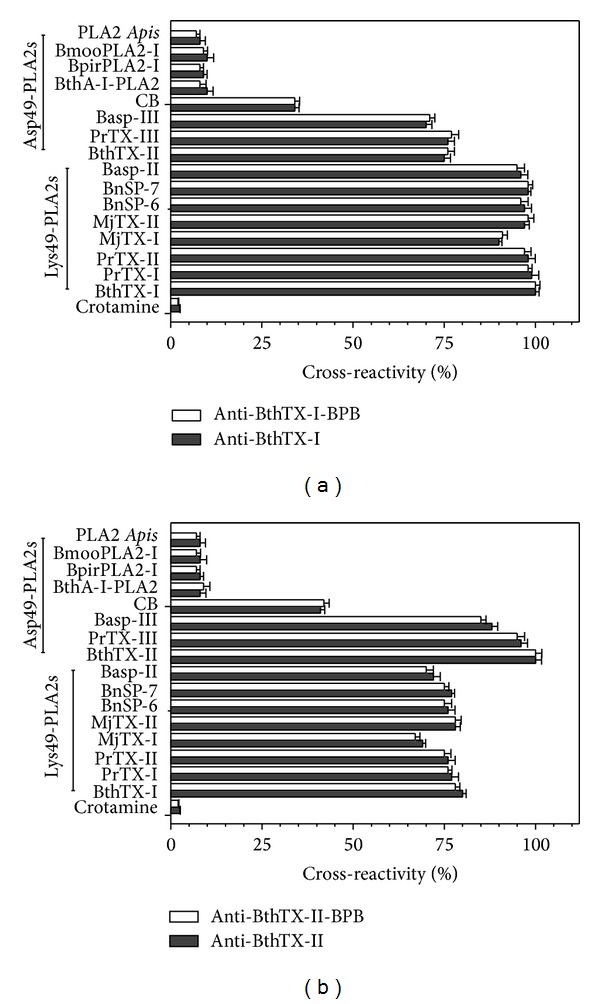
Cross-reactivity of antibodies produced from native or alkylated proteins BthTX-I and BthTX-II (anti-BthTX-I, anti-alk-BthTX-I, anti-BthTX-II, and anti-alk-BthTX-II) against other isolated PLA_2_s, as shown by enzyme immunoassay. Lys49-PLA_2_ homologs (*Bothrops jararacussu* BthTX-I,* B. pirajai* PrTX-I and II,* B. moojeni* MjTX-I and II,* B. pauloensis* BnSP-6 and 7, and* B. asper* Basp-II), basic Asp49-PLA_2_s (*B. jararacussu* BthTX-II,* B. pirajai* PrTX-III,* B. asper* Basp-III, and* Crotalus d. terrificus* CB), acidic Asp49-PLA_2_s (*B. jararacussu* BthA-I-PLA_2_,* B. pirajai* BpirPLA_2_-I, and* B. moojeni* BmooPLA_2_-I), and PLA_2_ from* Apis mellifera* venom [2–4, 6, 21, 30, 45, 46]. Microplate wells were coated with antigen, and antibody binding was measured, as described in Section 2.5.2. Cross-reactivity was expressed as a percentage of the absorbance signal resulting from the binding of antibodies to the homologous antigen BthTX-I or -II. Crotamine was included as an unrelated, negative control antigen. Results expressed as means ± S.D. (*n* = 3).

Our results demonstrate that the alkylated PLA_2_ has the capacity to produce antibody. To verify the efficiency of these polyclonal antibodies produced to recognize the venoms of snakes belonging to the genus* Bothrops spp.*, we purified these antibodies: anti-BjussuCV, anti-alk-BjussuCV, anti-BthTX-I, anti-BthTX-II, anti-alk-BthTX-I, and anti-alk-BthTX-II. They were then incubated (30 min at 37°C) with total venom before administration. In addition, we also extended our studies to check whether the polyclonal antibodies had the capacity to recognize the venoms of* Crotalus spp.* and* Micrurus spp*. (Figures 2(a)–2(c)). To increase the range of our results, we also analyzed the capacity of the polyclonal antibodies to recognize PLA_2_s isolated from a variety of venoms. The antibodies were incubated with PLA_2_ before inoculation (30 min at 37°C) (Figures 3(a) and 3(b)). All enzymes and myotoxins were isolated as previously reported [2–4, 6, 21, 30, 45, 46], and PLA_2_ from* Apis mellifera* was purchase from the Sigma-Aldrich (PN P9279, CAS 9001-84-7, Missouri, USA).

Myotoxic activities of BjussuCV were reduced after incubation with anti-BjussuCV serum. The myotoxic activity of the isolated toxins was also reduced by the antibodies produced against native and modified toxins (anti-BthTX-I, anti-BthTX-II, anti-alk-BthTX-I, and anti-alk-BthTX-II), as well as crude venom (anti-BjussuCV and anti-alk-BjussuCV). The phospholipasic activity of BjussuCV decreased after incubation with anti-BjussuCV serum and, to a lesser extent, with anti-alk-BjussuCV serum (Table 2).

All antibodies neutralized the lethality of the venoms. No mouse died when treated with anti-BjussuCV, anti-alk-BjussuCV, anti-BthTX-I, or anti-BthTX-II, and the mortality was reduced by around 83% in response to anti-alk-BthTX-I and anti-alk-BthTX-II.

**Figure 4:**
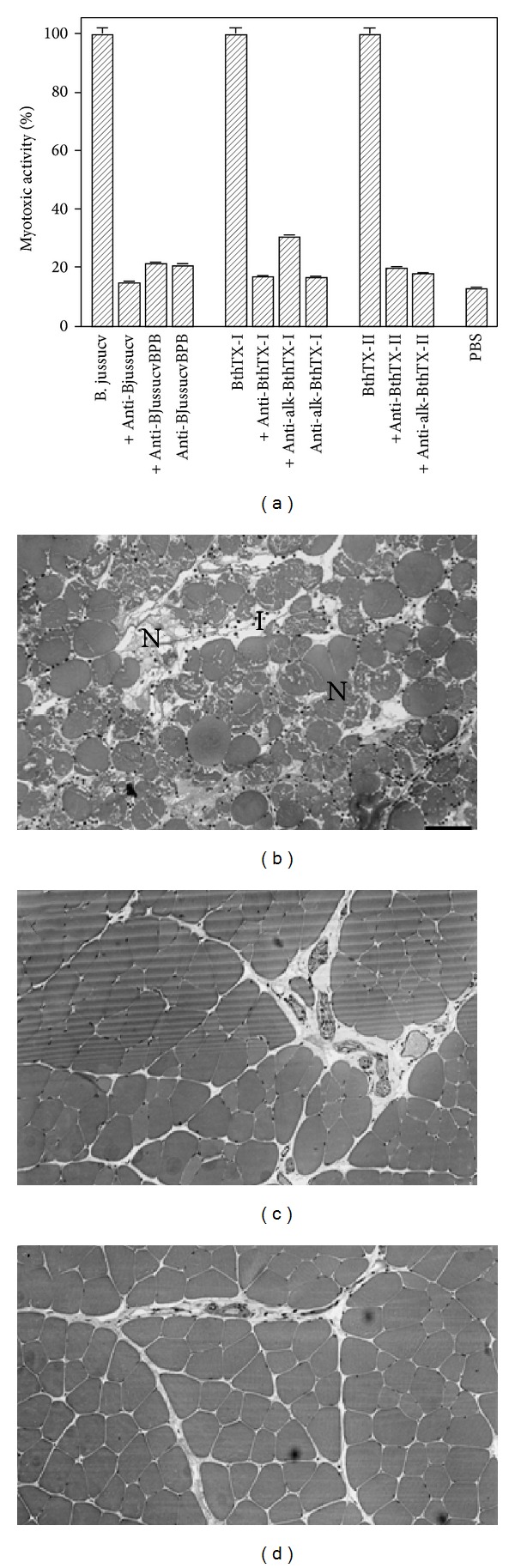
Inhibition of the myotoxic activity by antibodies against native and BPB-modified crude venom and myotoxins. (a) Myotoxic effect of crude venom or isolated PLA_2_ alone or following incubation with antibodies (50 : 1 antibody : toxin, w/w) in mice. The values were estimated by the levels of creatine kinase (CK) activity in plasma 3 h after the injection of samples. Phosphate-buffered saline (PBS) was included as a control. Histopathological analysis induced by myotoxins. Light micrographs of sections of mouse gastrocnemius muscle 24 h after injection of 50 *μ*g of myotoxins (BthTX-I or BthTX-II) alone (b) or incubated with antibodies (50 : 1 antibody : toxin, w/w), dissolved in 50 *μ*L PBS, and stained with hematoxylin and eosin. (c) Control mice were injected with PBS alone; (d) normal integer fibers are seen. Note the presence of myonecrosis (N) and an abundant inflammatory infiltrate (I).

Only 15% of the edema-inducing activity was reduced in the presence of anti-BthTX-II, while anti-BthTX-I did not induce any significant reduction in this activity (only 5%), thereby indicating that the site responsible for myotoxicity (Figure 4) differs from that responsible for edema-inducing activity. Experiments addressing the phospholipasic activity showed that antibodies against the native and alkylated venom neutralized about 80% and 50% of the phospholipasic activity, respectively (Table 1).

The myotoxic effect of the crude venom or myotoxins, with or without incubation with antibodies, was estimated by the levels of CK activity in plasma 3 h after injection of the samples. Both native and alkylated antibodies reduced the myotoxic activity induced by the crude venom or isolated PLA_2_ (Figure 4(a)). Furthermore, the inhibition of the myotoxicity induced by the toxins was clearly observed after histopathological analysis of the mouse gastrocnemius muscle sections obtained 24 h after injection of PBS (Figure 4(d)), 50 *μ*g of PLA_2_ (BthTX-I or BthTX-II) (Figure 4(b)) or PLA_2_ previously incubated with the antibodies (Figure 4(c)).

**Figure 5:**
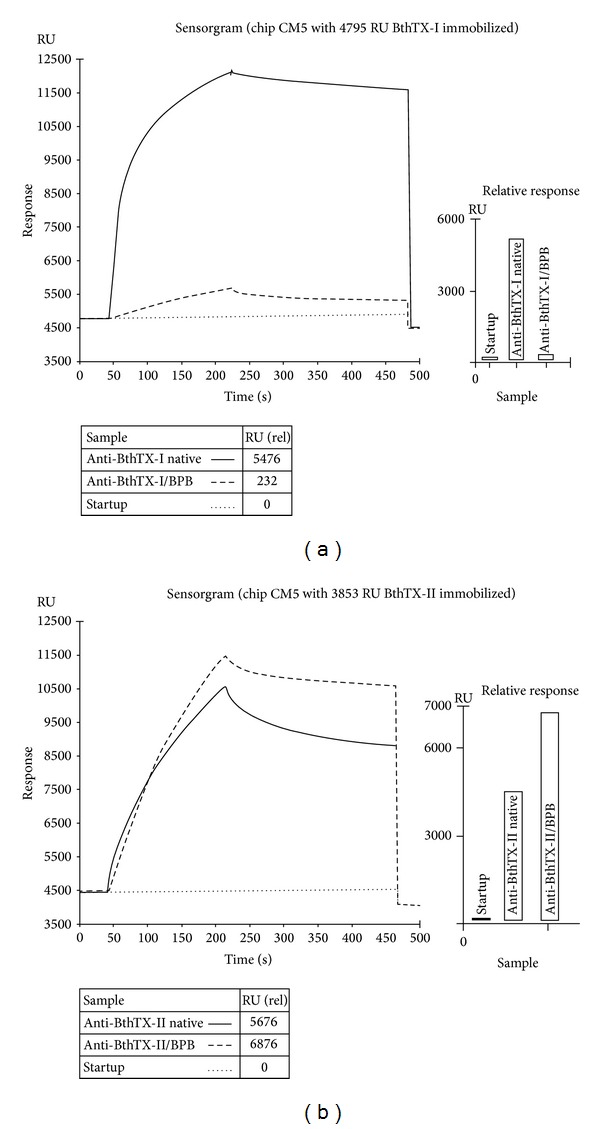
SPR showing protein interactions with immobilized BthTX-I (a) and BthTX-II (b) assay with native antibodies and antibodies chemically alkylated by* p*-bromophenacyl bromide (BPB). Concerning antibodies for the native and alkylated BthTX-I and BthTX-II, the assay was conducted at a flow rate of 30 *μ*L/min for 1 min at 37°C. These samples were carried with HBS-P 1X Buffer (GE Healthcare, USA) pH 7.4.

SPR data showed a loss of the affinity [39] for anti-BthTX-I/BPB in comparison with the native anti-BthTX-I for interaction with BthTX-I (Figure 5(a)) but anti-BthTX-I/BPB showed high affinity for immobilized BthTX-II when compared with native anti-BthTX-I (Figure 5(b)) [44]. These results reinforce the antigenicity results revealed in the ELISA assays.

## 4. Discussion and Conclusion

The design of immunization experiments varies depending on the origin of the antigen and the method of inoculation. Here, we immunized Swiss mice through repeated subcutaneous injections of a fixed dose of the antigen with an interval of 15 days. At the end of 6 weeks, “proof bleeding” was done, and an immunodiffusion test was performed to check the presence of antibodies. The final bleed was then carried out. Immunoglobulin G was initially purified by precipitation of the total globulin fraction and subsequently by ion exchange chromatography on DEAE-Sephacel, leading to a high yield of purified product (Figure 1(a)).

The immunodiffusion test demonstrated that sufficient levels of antibodies were produced after the administration of 2 and 3 doses of the antigen, myotoxins, and crude venom, respectively. Our results show that the toxins, despite modification, did not lose their antigenicity (Figure 1(b)). In an analogous strategy, Soares et al. [28] described similar findings, but only with a basic Lys49-PLA_2_-like protein and crude venom from* B. moojeni*.

Immunodiffusion of the toxins and rabbit plasma aliquots demonstrated the formation of precipitation lines in all cases. This observation indicates that the covalent modification of the His48 residue did not interfere with the structure of the proteins, thereby not diminishing their antigenicity. The same experiment was carried out with the antibodies against native and alkylated crude venom (Figure 1(c)). Immunoassays (ELISA) were performed to evaluate the specificity of the antibodies for their respective antigens. The antibodies recognized the BthTX-I and -II proteins (PLA_2_) indistinctly. For the antibodies against BjussuCV, venoms from snakes of the Crotalidae (*B. moojeni, B. asper, B. neuwiedi, B. pirajai,* and* C. d. terrificus*) and Elapidae (*Micrurus frontalis*) families were used to test cross-reactivity. Results showed a cross-reactivity of about 100% for Crotalidae venoms, while, for Elapidae venoms, this cross-reactivity was about 70%. De Roodt et al. [49] reported that the high cross-reactivity found between venoms and various antivenins supports the use of antigens with common epitopes to induce the production of antibodies with neutralizing potential against various toxic proteins.

These results are consistent with data of the cross-reaction obtained with antivenoms produced against Crotalidae venoms, which neutralize several biological activities of other venoms belonging to snakes of the same or another family (Viperidae) [50]. This cross-reactivity occurs as a result of the high degree of similarity in the structure of homologous proteins, such as the PLA_2_s of taxonomically related species [51].

Studies show that a “family” of antigenically-related myotoxic PLA_2_ occurs in many types of venom of the* Bothrops* genus (Figure 4). Conservation of at least some epitopes in several PLA_2_s, which is in accordance with their high homology, both in amino acid sequences and tridimensional structure, was also discussed [2, 28, 52]. Furthermore, SPR data (Figure 5) revealed the conservation of antigenicity; if, on one hand, anti-BthTX-I/BPB loses affinity for immobilized BthTX-I, the same anti-BthTX-I/BPB gains affinity for immobilized BthTX-II. These findings were verified by an affinity curve and by sum of mass for protein immobilized affinity [53].

The lethality assay with native and alkylated BjussuCV antivenoms, capable of neutralizing 2 × LD_50_, resulted in 100% survival of the animals (Table 2). The antibodies recognized the antigens and completely neutralized their activity. In contrast, the antibodies against alkylated BthTX-I and -II led only to partial neutralization and only 17% of the animals survived. These results suggest a low affinity between antigen and antibody and the need to increase the amount of antibody to achieve greater neutralization or even a loss of antigenic response.

For antibodies against the native and alkylated venom, inhibition assays were performed to examine the phospholipasic, edema-inducing, and myotoxic activities. Antibodies produced against the alkylated venom showed high inhibitory activity (50–100%) against PLA_2_ activities, resulting in inhibition values closer to those obtained with the antibodies against the native crude venom. These results indicate that the loss of enzymatic activity of some venom toxins does not affect their immunogenicity and point to the possibility to produce identical antibodies against native and alkylated venoms or even different antibodies able to recognize the epitopes of the native and alkylated toxins.

Another experiment demonstrated that the antibodies against the native venom inhibited about 15% of edema-inducing activity. This finding corroborates the results of previous studies that reported the inefficiency of antivenom in inhibiting this activity [54, 55] since the victim's organism reacts against unknown molecules, leading to an inflammatory response. Local tissue damage (myonecrosis, dermonecrosis, and edema) is a serious consequence of the envenomation by snakes of the genus* Bothrops*. The animals used to produce anti-serum present the same damage at the sites of injections [2–5]. The pathogenesis of these alterations is complex, since these activities are induced by several toxins present in the venom [56, 57].

We performed PLA_2_ activity assays only with the catalytically active isoform (BthTX-II-D49PLA_2_). This activity was neutralized by about 50% by antibodies against the native toxin and by about 15% by antibodies against alkylated PLA_2_. Lomonte et al. [58] demonstrated the capacity of antibodies against* B. asper* myotoxin to neutralize the phospholipase activity of myotoxin I of the same venom. This cross neutralization shows that there are conserved epitopes of relevance to the structure of these PLA_2_ isoforms. We observed that neutralization of the myotoxic activity induced by the native venom, as determined by the CK levels, was about 80% for all antibodies raised against both BthTX-I and II. These results show that the basic myotoxins are key factors responsible for the toxicity of* B. jararacussu* venom [27].

Several structural studies have reported that only the His48 residue is chemically modified by BPB [23, 27, 28, 55]. This alkylation occurs in the imidazole ring (N*δ*1 atom) of the His48 residue present in the active site and promotes the loss of PLA_2_ enzymatic activity and/or reduction of the toxic and pharmacological effects of this molecule [23]. These findings suggest that these pharmacological effects and the catalytic activity are dependent on His48. The Lys49-PLA_2_s are bounded to BPB and showed a decrease in myotoxic activity of around 40–50%, a decrease in cytotoxicity of around 85–90%, and a 15–20% decrease in capacity to induce edema. Also, Lys49-PLA_2_s are bounded to BPB and significantly reduces mortality, without any significant change in the liposome-disrupting capacity, thereby also suggesting a dependence of these pharmacological effects and the “active site” of these proteins [59].

In the case of Lys49-PLA_2_s, several studies have identified the C-terminal region as responsible for their cytotoxicity and other toxicological activities [60]. A specific myotoxic site formed by the Lys20, Lys115, and Arg118 residues was proposed for snakes of the* Bothrops* genus [61]. The preliminary crystal structure of BthTX-I chemically modified by BPB suggests that the binding of the inhibitor leads to significant structural modifications, especially in the C-terminal region [62].

**Figure 6:**
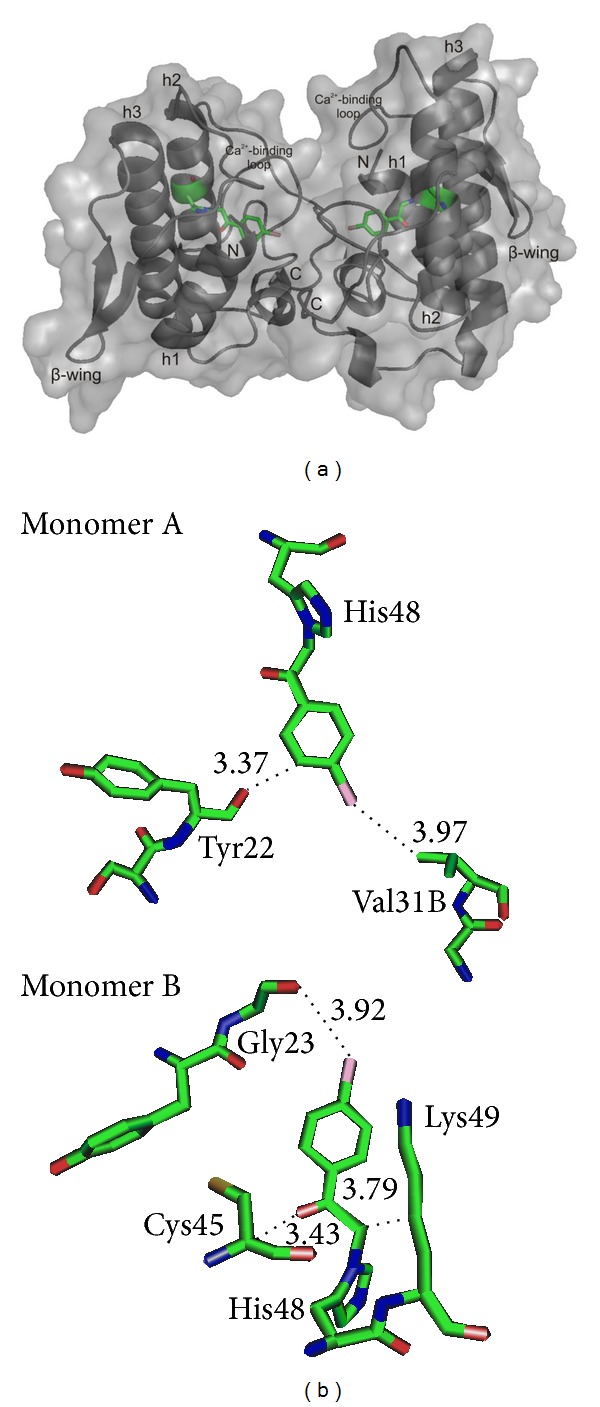
Preliminary crystal structure of BthTX-I chemically modified by* p*-bromophenacyl bromide (BPB). (a) Ribbon representation of BthTX-I/BPB structure with the secondary structure elements marked: the Ca^2+^-binding loop (25 to 34), *β*-wing (75 to 84), and the three *α*-helices—“h1” (1 to 12), “h2” (40 to 58), and “h3” (89 to 110). The* p*-bromophenacyl bromide molecules are highlighted as green sticks. (b) Top view of BPB covalently bound to the His48 residue of the “active site” region of monomers A and B of the BthTX-I/BPB complex. Interactions of BPB with the protein residues are indicated. The sequences have been numbered following Renetseder and coauthors [47]. Molecules were drawn using the PyMOL program [48].

In this structure, the phenacyl group of the BPB molecule extends along the hydrophobic channel of the protein, interacting with Tyr22, Gly23, Val31, Cys45, and Lys49 residues (Figure 6).

Recently, the structure of Lys49-PLA_2_, following PrTX-I chemical modification by BPB, was resolved by X-ray crystallography [59]. This protein shares 98% similarity with BthTX-I and thus could be an interesting model for the BthTX-I-BPB structure. BPB is a small molecule that covalently binds to the His48 residue but retains the capacity to distort the Ca^2+^-binding loop region, rearranging the C-terminus as a result of the establishment of a new interchain hydrogen bond between the Tyr119 residues [60]. This rearrangement of the C-terminus induces an alignment between the monomers, changing the quaternary conformation of the PrTX-I/BPB structure [60, 62]. These data, as previously observed Nomura et al. [32], suggest the presence of two distinct binding regions of Lys49-PLA_2_s on membranes: the “active site/hydrophobic channel” and the “C-terminal/myotoxic site.”

Our results indicate that the chemical modification of the myotoxins attenuate their toxic potential but do not change their antigenicity. For the native venom, the toxicity was partially reduced and the antigenicity remained unaltered when the venom was modified with BPB.

Taken together, these data demonstrate the high potential of the use of chemical modification to reduce venom toxicity [63]. These studies provide an alternative protocol for increasing the amount of venom used to vaccinate horses in order to obtain higher antibody titers in the antiophidic serum. These toxins are the main factors responsible for local damage in bothropic envenomation. Thus, it may be possible to increase the titer of antimyotoxin antibodies in antiophidic serum without major complications in the immunized animals.

## Figures and Tables

**Table 1:** Influence of BjussuCV, BthTX-I, BthTX-II, alk-BjussuCV, alk-BthTX-I, and alk-BthTX-II on LD_50_ and phospholipase A_2_ (PLA_2_) activity.

Sample	LD_50_ (mg/kg)	PLA_2_* (U/mg)
BjussuCV	5.7 ± 0.9	71.9 ± 1.1
Alk-BjussuCV	5.8 ± 0.8	Residual
BthTX-I	8.0 ± 0.9	0.0
Alk-BthTX-I	40.0 ± 1.2	0.0
BthTX-II	7.5 ± 0.8	37.1 ± 1.2
Alk-BthTX-II	37.3 ± 1.1	0.0

*Measured through potentiometric titration [37].

**Table 2:** Neutralization of edema, PLA_2_ activity, and 2x LD_50_ induced by the respective antibodies of native and modified forms of* B. jararacussu* crude venom and isolated toxins.

Venom or isolated PLA_2_	Neutralization of 2 × LD_50_*		Neutralization of edema	Neutralization of PLA_2_ activity	
	Antibodies		Antibodies	Antibodies	
	Native	Alkylated	Native	Native	Alkylated
BjussuCV	100%	100%	30%	80%	50%
BthTX-I	100%	83%	5%	—	—
BthTX-II	100%	83%	15%	55%	25%

*LD_50_ assay was done after previous incubation of the venom or toxin with its respective antibody (1 : 50, w/w) for 30 min at 37°C. Results express the percentage of animal survival.

## Abbreviations

PLA_2_: Phospholipase A_2_
BjussuCV: * Bothrops jararacussu* crude venom
BthTX-I: Phospholipase A_2_ Lys49 from* B. jararacussu*venom
BthTX-II: Phospholipase A_2_ Asp49 from* B. jararacussu*venom
BPB: * p*-Bromophenacyl bromide
Anti-BjussuCV: Antibodies against* B. jararacussu* crude venom
Anti-BthTX-I: Antibodies against* B. jararacussu* myotoxin BthTX-I
Anti-BthTX-II: Antibodies against* B. jararacussu* myotoxin BthTX-II
Anti-BjussuCV-BPB: Antibodies against* B. jararacussu* BPB modified crude venom
Anti-BthTX-I-BPB: Antibodies against* B. jararacussu* myotoxin BPB modified BthTX-I
Anti-BthTX-II-BPB: Antibodies against* B. jararacussu* myotoxin BPB modified BthTX-II.
